# Homozygote Depression in Gamete-Derived Dragon-Fruit (*Hylocereus*) Lines

**DOI:** 10.3389/fpls.2017.02142

**Published:** 2018-01-05

**Authors:** Daqing Li, Maria F. Arroyave Martinez, Ruth Shaked, Noemi Tel-Zur

**Affiliations:** ^1^French Associates Institute for Agriculture and Biotechnology of Drylands, The Jacob Blaustein Institutes for Desert Research, Ben-Gurion University of the Negev, Beersheba, Israel; ^2^College of Agriculture and College of Life Science, Guizhou University, Guiyang, China

**Keywords:** breeding, gametoclonal variation, gamete-derived progenies, perennial fruit crop, sterility, vine cacti

## Abstract

Putative gamete-derived progenies from two *Hylocereus* species, the diploid *H. monacanthus* and the tetraploid *H. megalanthus*, were studied with the dual aims to confirm their gamete origin and to evaluate their potential use as genetic resources. An additional goal was to determine the origin (allotetraploid vs. autotetraploid) of *H. megalanthus* by exploring morphological variations in the di-haploid (2*x*) *H. megalanthus* progeny. Gamete origin was proved in all five *H. monacanthus* lines obtained and in 49 of the 70 *H. megalanthus* lines by using flow cytometry and simple sequence repeat (SSR) markers. The five double-haploid (2*x*) *H. monacanthus* lines showed low vigor and abnormal flower development, with malformed ovules and aborted pollen grains. Only one flower set fruit, giving several viable seeds. For *H. megalanthus*, both abnormal ovules and defective anthers were observed in the di-haploid (2*x*) and double di-haploid (4*x*) lines. Among the 46 di-haploid lines, only 14 set fruit. Another 13 di-haploid lines formed flower buds that abscised before anthesis or soon after pollination. The severe sterility of the double-haploid *H. monacanthus* and the reduced fertility of all the di-haploid and double di-haploid *H. megalanthus* lines can be linked to their reduced heterozygosity, which drastically affected the development of normal female and male organs. We thus concluded that chromosome doubling, as occurred spontaneously in the double-haploid *H. monacanthus* and the double di-haploid *H. megalanthus*, is not sufficient to restore fertility in *Hylocereus*. We also observed very low gametoclonal variation among the di-haploid (2*x*) *H. megalanthus* lines, a finding that supported an autotetraploid, rather than an allotetraploid, origin of this species. Nonetheless, despite the above-described challenging limitations, these gamete-derived lines are currently being bred as the seed parent, offering unique possibilities for genetic research and additional breeding.

## Introduction

The genus *Hylocereus* (Cactaceae) of the vine cacti, known as dragon fruit or pitayas, comprises 15 perennial species (Bauer, 2003). These *Hylocereus* species bear medium to large (200–800 g) edible fruits with broad scales, various peel and flesh colors, and numerous small soft seeds (Tel-Zur et al., 2004a, 2011b). Among these species, those cultivated worldwide include: the diploids *H. undatus* and *H. monacanthus*, which have white, purple or red flesh and a red peel; the tetraploid *H. megalanthus*, with white flesh and a yellow spiny peel (Lichtenzveig et al., 2000); and the spontaneous (natural) or induced (artificial) interspecific hybrids of these three species (Tel-Zur et al., 2012a; Pagliaccia et al., 2015). Interest in these species has increased markedly in recent years, since they are regarded as exotic fruit crops with high economic potential and as a rich source of natural micronutrients (Wu et al., 2006) and phytochemicals with therapeutic activities (Song et al., 2016). This rise in interest is indeed reflected in the registration of many new cultivars in recent years (Lobo et al., 2016). An important plant characteristic promoting the expansion of areas under cultivation with these species is their crassulacean acid metabolism (CAM), which confers exceptional tolerance to extreme drought: such plants show four- to six-fold higher water use efficiencies than other fruit crops grown under similar conditions (Mizrahi et al., 2007). These cactus species are thus a valuable commodity for dryland farmers in regions with increasing water scarcity and land degradation.

Despite the growing interest in these fruit crops, they remain underutilized minor perennial crops, and there is a need for effective breeding programs to facilitate the expansion of areas under cultivation. To this end, a long-term breeding program was begun some three decades ago at Ben-Gurion University of the Negev (BGU) to develop improved hybrids with superior fruit quality, good yields, and enhanced tolerance to extreme temperatures (Tel-Zur, 2013). However, this breeding program—like many breeding programs for other minor crops—has been hampered by a variety of factors, including difficulties in applying molecular and other updated breeding approaches, the limited genomic information currently available, and the plants' large genome size and high heterozygosity (Cisneros and Tel-Zur, 2013; Plume et al., 2013; Pagliaccia et al., 2015). In addition to all these factors, 6–10 generations of self-pollination are needed to obtain phenotypically stable homozygote lines. Since *Hylocereus* species are perennial species with a prolonged juvenile stage and a strong self-incompatibility system in the diploid species, the use of both traditional and advanced breeding approaches faced severe limitations. There was thus a need to develop the accurate platform research required to breed these crops.

To meet this need, the first step in the BGU breeding program was an investigation of the taxonomy and ploidy of the *Hylocereus* species. The original classification of *H. megalanthus* was performed by Britton and Rose (1920) on the basis of its unique morphology: since the species has a triangular stem, like that of all *Hylocereus* species, and spiny fruits, like those of *Selenicereus*, they classified it into a separate genus, which they named *Mediocactus*, thereby implying both an intermediate morphology and an intermediate taxonomic status. Nonetheless, Bauer has placed this species in the *Hylocereus* genus, reflecting the close affinity between this tetraploid species and the other species of this genus, which are all diploids (Bauer, 2003; Tel-Zur et al., 2011b). The fact that a tetraploid taxon shares morphological features with two genera (*Hylocereus* and *Selenicereus*), which are also cross compatible (Lichtenzveig et al., 2000; Tel-Zur et al., 2012b), might imply an allopolyploid origin. Indeed, early studies suggested an intermediate taxonomic status for *H. megalanthus* (Tel-Zur et al., 2004a,b), but no evidence of an allopolyploid origin was found in later work (Plume et al., 2013). Thus, the origin of *H. megalanthus* remains an open question—one that can perhaps be resolved taking an approach that differs from the more conventional methodologies described above, namely, by applying the idea of “gametoclonal variation” put forward by Morrison and Evans (1987) to describe the variation observed among plants regenerated from gamete cells in culture. The regeneration of *H. megalanthus* plants with half a set of chromosomes (two instead of four) via anther or ovule culture may offer an additional tool to explore the origin of this tetraploid species.

In both *H. megalanthus* and *H. monacanthus*, successful haploid induction via androgenesis or gynogenesis (Garcia et al., 2009a,b) has paved the way for breeding programs and genetic studies, since gamete-derived progenies have, theoretically, a lower level of heterozygosity than that of the parental lines (Verdoodt et al., 1998). Moreover, favorable outcomes may be expected from haploid induction, since polymorphism, at the molecular and phenotypic levels, between the parental line and the gamete-derived progenies will lead to the creation of novel phenotypes due to homozygous recessive genes and gametoclonal variation (Morrison and Evans, 1987). These outcomes will facilitate a search for molecular markers related to agronomic traits for plant improvement (Germanà, 2011; Ravi et al., 2014).

Here, we report a comprehensive evaluation of the gamete-derived progenies from *H. monacanthus* and *H. megalanthus* that were previously generated by Garcia et al. (2009a,b). On the basis of a previously reported correlation between ploidy level and stomatal density for a number of species (Beaulieu et al., 2008) and also for *Hylocereus* (Tel-Zur et al., 2011a,b), we performed a comparative stomatal study between the donor plants and the putative gamete-derived lines to evaluate the utility of applying this tool as a preliminary screen for ploidy. We then confirmed the ploidy level by using flow cytometry. In parallel, we proved the gamete origin of these lines by using simple sequence repeat (SSR) markers at the sequencing level. We also evaluated the gamete-derived progenies in terms of morphological traits, pollen viability, ovule development, fruit weight and reproductive potential in comparison with the donor species. In addition to reporting these findings, in this article we also address gametoclonal variation in the regenerated di-haploid *H. megalanthus* lines, thereby shedding light on the possible origin of this tetraploid species. Finally, we discuss the value of these lines for further genomic analysis and breeding programs.

## Materials and methods

### Plant material and growing conditions

Gamete-derived progenies were obtained through the induction of androgenesis and gynogenesis (Garcia et al., 2009a,b) in the framework of the BGU breeding program. Five putative androgenic lines from *H. monacanthus* (accession 89-028) and 70 putative androgenic and gynogenic lines from *H. megalanthus* (accessions 90-002 and 96-663) were generated in 2009, and ever since then the plants of these lines have been cultivated in pots in our greenhouse on BGU's Sede Boqer Campus (30°51′8″N/34°47′0″E). Water mixed with fertilizer (70 ppm N, 9 ppm P, and 70 ppm K) is routinely applied by drip irrigation all year round, with each plant being irrigated daily with 0.6 l. The average day/night temperatures in the greenhouse are 28/12°C during the cold season and 35/18°C in the hot season.

### DNA ploidy determination using flow cytometry analysis

The nucleus extraction process and flow cytometry analysis were performed as described in Li et al. (2017). The flow cytometer used was an iCyt SY3200 sorter (Sony Biotechnology, San Jose, USA) equipped with an 810-nm laser and a 792/50 band-pass filter for the forward scatter channel (FSC) and a 561-nm laser with a 605/70 band-pass filter for the PI channel. DNA ploidy of the gamete-derived progenies was assessed by comparing their nuclear DNA content with that of the donor plants. Each plant was analyzed at least three times to verify reproducibility of the results, and the standard deviation (sd) was calculated.

### Confirmation of the gamete origin using SSR markers

Genomic DNA was isolated from epidermis tissues according to Allen et al. (2006) with an adjusted CTAB buffer [0.05 M CTAB, 20 mM EDTA, 100 mM Tris-HCl (pH 8.0), 3 M NaCl, and 1% polyvinylpyrrolidone (PVP-360) (w/v) added before use]. Since species-specific SSR primers have not yet been developed for *Hylocereus* species, a total of 23 pairs of SSR markers previously reported for other Cactaceae were used (Table S1). The PCR reaction was set up as described by Cisneros and Tel-Zur (2013), and 10 μl of PCR products were separated in non-denaturing 6% polyacrylamide gels. DNA bands were stained with 0.5 mg/ml ethidium bromide and photographed using a G-box under UV light. Allelic bands showing polymorphism between the donor plant and the gamete-derived lines were isolated from the gel, as described by Sambrook and Russell (2006), and sequenced. For DNA sequencing, a CloneJET PCR cloning kit (Thermo Scientific, cat. no. K1231) was used for the blunting and ligation steps, according to the protocols supplied by the manufacturer. Ligation mixtures were transformed using a TransformAid bacterial transformation kit (Thermo Scientific). A single isolated colony from a selective LB agar medium (supplemented with 200 μg/ml ampicillin) was picked out for overnight bacterial culture. Plasmid DNA isolation was performed using the alkaline lysis method (Birnboim and Doly, 1979). To confirm transformation success, pJET1.2 forward and reverse primers (Thermo Scientific) were applied, followed by band checking using electrophoresis.

All the sequences obtained from the SSR locus were compared against the sequences deposited in GenBank, using the BLAST programs (http://www.ncbi.nlm.nih.gov/BLAST/), and the similarities were calculated.

### Cytological, histological, and morphological evaluation

Measurements of the density and length of the stomata were performed for all the putative gamete-derived *H. monacanthus* and *H. megalanthus* lines by using the silicon rubber imprints technique (Smith et al., 1989). Flower bud morphology and size at anthesis were recorded for all the putative gamete-derived *H. megalanthus* lines. Flower length was measured with a measuring tape a few hours before anthesis. Flower width at anthesis was measured with a caliper at the widest part of the ovary. At anthesis, 19 and 7 flowers from the gamete-derived and the somatic tetraploid lines, respectively, were measured. Fruits were harvested at full maturation and weighed using a laboratory balance. A total of 186 mature fruits was weighed; 98 from the gamete-derived lines and 88 from the somatic tetraploid regenerants.

Pollen viability was assessed for all the putative gamete-derived *H. megalanthus* lines by staining fresh pollen collected at anthesis with 2% w/v aceto-carmine. This staining agent was chosen in light of our previous work showing it to be as suitable as other staining agents, such as Alexander's Schiff reagent and fluorescein diacetate, for staining pollen grains (Tel-Zur et al., 2005).

Pollen grains with a typical subglobular shape and strong stainability were scored as viable, while weakly stained grains and/or those with an atypical shape were scored as aborted. At least 300 pollen grains from three different flowers per plant were scored.

Androgenic *H. monacanthus* flower buds dropped off at early flower development. We therefore studied ovule and anther development during several developmental stages. Since we knew from our previous work (Lichtenzveig et al., 2000) that meiosis occurs in the donor plant *H. monacanthus* when the flower bud reaches 5.0–5.5 cm in length, we collected flower buds for histological studies at early flower bud development (1.0–1.5 cm length), following meiosis (5.5–6.0 cm length) and at late development (12.0–12.5 cm). Tissue fixation, embedding, sectioning and Harris hematoxylin and eosin-Y staining protocols were performed as described in Cisneros et al. (2011). Slides were examined through an Axio ImagerA1 microscope with LED illumination (Zeiss) and photographed with an AxioCam HRC camera (Zeiss).

### Statistical analysis

For the *H. monacanthus* lines and for flower morphology and fruit weight in *H. megalanthus* lines, we performed one-way ANOVA using IBM SPSS Statistics software version 22 (IBM Corp., Armonk, NY, USA), and pairs of means were compared by a Tukey HSD test (*P* < 0.05). For the comparisons of mean parametric groups for stomatal density and length in putative-gamete derived *H. megalanthus* lines, data was analyzed using unpaired *t*-test with Welch's correction.

## Results

### Ploidy level estimation using flow cytometry

The mean nuclear 2C DNA of the parental species, namely, the diploid *H. monacanthus* and the tetraploid *H. megalanthus*, were previously reported to be 3.9 and 8.6 pg, respectively (Tel-Zur et al., 2011b). The genome sizes of the putative gamete-derived progenies were evaluated and compared with those of their donor species. For *H. monacanthus*, no statistically significant differences were observed between the nuclear DNA content of the donor parent and that of the five androgenic lines (Table 1). Since Garcia et al. (2009b) had previously reported a haploid status for these androgenic *H. monacanthus* lines during early development, we assumed that spontaneous chromosome doubling had subsequently occurred, and thus SSR markers were used to confirm the gamete origin of these five lines (see below).

**Table 1:** Nuclear DNA content, stomatal density and length, and DNA ploidy estimation in five *H. monacanthus* androgenic lines.

**Donor species or androgenic line code**	**Nuclear DNA content (pg/2C ± SD)**	**Stomatal density (number per mm^2^ ± SD)**	**Stomatal length (μm ± SD)**	**DNA ploidy estimated**
H. monacanthus^*^	3.89 ± 0.13^a^	13.11 ± 0.69^c^	58.87 ± 2.32^a^	2x
007	3.83 ± 0.14^a^	13.17 ± 1.28^c^	56.05 ± 4.58^ab^	2x
008	3.7 ± 0.19^a^	16.2 ± 1.71^ab^	55.11 ± 6.96^ab^	2x
010	3.79 ± 0.16^a^	15.44 ± 1.72^b^	53.48 ± 6.18^b^	2x
011	3.96 ± 0.19^a^	16.67 ± 2.46^a^	54.43 ± 3.12^b^	2x
7057	3.63 ± 0.25^a^	17.48 ± 2.6^a^	48.05 ± 3.42^c^	2x

* *Nuclear 2C DNA of the donor species, H. monacanthus accession 89-028, was previously reported by Tel-Zur et al. (2011b)*.

*Values with different superscript letters are significantly different (P < 0.05)*.

For *H. megalanthus*, plants of 46 of the 70 putative gamete-derived lines studied showed half (or close to half) the nuclear DNA content compared to the tetraploid donor species, thus confirming their gamete origin (Table 2). For three lines, 432, 1347, and 1736, the nuclear DNA content was similar to that of the donor species, but their atypical morphology and phenology suggested a gamete origin, which was subsequently confirmed using SSR markers (see below).

**Table 2:** Nuclear DNA content, stomatal density and length, pollen viability, fruit weight and DNA ploidy estimation in putative gamete-derived lines from the tetraploid donor *H. megalanthus* (accessions 90-002 and 96-663).

**Line code**	**Nuclear DNA content (pg/2C ± SD)**	**Stomatal density (number per mm^2^ ± SD)**	**Stomatal length (μm ± SD)**	**Pollen viability (% ± SD)**	**Fruit weight (g ± SD)**	**DNA ploidy estimated**
90-002	8.57 ± 0.40^♦^	6.82 ± 2.11	64.8 ± 4.24	73.7 ± 1.46	130 ±52.5	4x
96-663	8.61 ± 0.28	6.99 ± 1.75	78.5 ± 2.61	70.2 ± 3.58	143 ±57.6	4x
5	3.77 ± 0.11	17.19 ± 2.21	50.14 ± 8.07	ND	ND	2x
6	4.08 ± 0.22	18.18 ± 2.28	66.81 ± 3.48	ND	ND	2x
8	3.94 ± 0.15	14.61 ± 2.59	65.13 ± 4.96	18.5 ± 3.16	112.0 ± 52.7	2x
9	4.41 ± 0.05	13.93 ± 1.17	65.26 ± 3.16	ND	ND	2x
19	4.33 ± 0.25	12.06 ± 1.61	57.16 ± 2.41	0	ND^**^	2x
23	4.09 ± 0.18	15.79 ± 2.62	66.33 ± 2.76	ND	ND	2x
26	4.41 ± 0.27	15.38 ± 1.68	65.39 ± 3.33	0	88.8 ± 83.6	2x
43	4.57 ± 0.07	14.57 ± 2.26	61.37 ± 4.85	3.69 ± 4.4	71.0 ± 64.8	2x
45	3.8 ± 0.15	12.88 ± 2.27	66.13 ± 3.77	0	ND^**^	2x
58	4.35 ± 0.26	15.09 ± 2.92	61.43 ± 6.53	ND	ND	2x
60	4.57 ± 0.16	16.55 ± 3.24	63.99 ± 3.22	ND	ND	2x
74	4.36 ± 0.12	14.1 ± 2.55	58.89 ± 4.42	0	ND^**^	2x
90	4.06 ± 0.19	14.22 ± 1.93	59.45 ± 5.87	ND	ND	2x
92	4.19 ± 0.21	14.63 ± 2.22	66.23 ± 2.39	0	ND^**^	2x
96	4.39 ± 0.14	15.44 ± 2.98	68.65 ± 3.28	ND	ND	2x
97	4.32 ± 0.05	14.68 ± 1.89	67.88 ± 2.47	0	ND^**^	2x
110	4.30 ± 0.24	16.67 ± 2.86	57.58 ± 3.17	0	151.6 ±70.7	2x
111	4.57 ± 0.12	16.32 ± 2.4	63.3 ± 4.14	0	ND^**^	2x
121	3.95 ± 0.11	13.69 ± 1.72	57.22 ± 2.87	0	57.6 ± 21.5	2x
122	4.11 ± 0.24	18.53 ± 2.85	58.39 ± 4.28	ND	ND	2x
124	3.97 ± 0.14	14.16 ± 1.4	57.59 ± 5.92	0	ND^**^	2x
125	4.22 ± 0.15	11.13 ± 1.34	64.29 ± 4.04	0	129.1 ± 88.0	2x
128	4.35 ± 0.15	16.84 ± 2.18	62.34 ± 4.88	0	ND^**^	2x
129	4.48 ± 0.23	13.64 ± 2.12	63.64 ± 3.27	0	ND^**^	2x
160	8.32 ± 0.19	5.59 ± 1.41	74.49 ± 4.46	68.77 ± 2.68	106.5 ± 34.9	4x
162	8.75 ± 0.28	6.24 ± 1.19	79.33 ± 3.86	ND	ND	4x
183	9.15 ± 0.31	5.94 ± 1.27	71.21 ± 3.04	70.51 ± 2.32	ND^**^	4x
199	8.04 ± 0.15	5.94 ± 1.35	70.18 ± 2.89	ND	ND	4x
201	8.61 ± 0.5	7.58 ± 1.91	73.31 ± 3.32	73.92 ± 1.97	96.65 ±19.6	4x
217	8.85 ± 0.41	6.18 ± 1.64	66.48 ± 3.67	ND	ND	4x
219	8.87 ± 0.47	8.33 ± 1.43	74.02 ± 2.8	ND	155.9 ± 123.7	4x
227	8.45 ± 0.42	5.89 ± 1.62	73.48 ± 3.98	ND	ND	4x
229	8.29 ± 0.09	6.64 ± 1.68	72.66 ± 5.06	78.49 ± 1.76	115.9 ±83.8	4x
235	8.56 ± 0.37	8.62 ± 2.55	69.35 ± 4.3	ND	ND	4x
244	8.76 ± 0.3	6.06 ± 1.7	65.07 ± 6.34	ND	ND	4x
247	8.48 ± 0.3	7.87 ± 1.7	73.69 ± 2.86	64.52 ± 3.65	151.5 ± 61.9	4x
264	8.28 ± 0.13	5.13 ± 1.65	73.22 ± 5.01	73.5 ± 0.84	ND^**^	4x
270	8.72 ± 0.08	6.18 ± 1.82	69.37 ± 2.88	74.43 ± 1.22	49.2 ± 27.6	4x
293	9.1 ± 0.14	6.06 ± 1.19	61.33 ± 6.68	70.38 ± 2.4	167.5 ± 78.2	4x
373	4.45 ± 0.2	15.15 ± 1.33	61.15 ± 1.9	ND	ND	2x
387	4.29 ± 0.26	26.86 ± 5.06	60.13 ± 4.62	0	61.3 ± 38.0	2x
388	3.79 ± 0.17	15.21 ± 1.73	61.03 ± 3.18	ND	ND	2x
422	4.35 ± 0.16	13.93 ± 2.81	38.98 ± 2.96	ND	ND	2x
423	4.5 ± 0.15	15.71 ± 2.11	56.73 ± 3.41	ND	ND	2x
425	4.25 ± 0.19	8.86 ± 1.83	59.87 ± 3.67	ND	ND	2x
426	3.97 ± 0.27	18.65 ± 2.49	57.35 ± 4.97	ND	114.5 ± 92.0	2x
427	4.52 ± 0.18	13 ± 1.7	63.17 ± 4.76	0	119.4 ± 72.0	2x
429	4.44 ± 0.04	16.49 ± 2.74	56.57 ± 5.52	8.16 ± 5.15	ND	2x
432	8.14 ± 0.21	6.99 ± 1.78	60.94 ± 4.47	9.81 ± 6.2	10.5 ± 0.5	4x
481	4.29 ± 0.22	11.71 ± 2.26	64.23 ± 3.92	12.58 ± 5.1	90.3 ± 40.6	2x
567	3.87 ± 0.1	12.24 ± 2.39	54.96 ± 2.73	0	30.6 ± 7.1	2x
569	4.40 ± 0.16	12.94 ± 2.32	57.26 ± 6.43	0	ND^**^	2x
1060	4.38 ± 0.13	14.28 ± 2.06	54.31 ± 6.05	4.26 ± 9.26	149.3 ± 97.0	2x
1341	4.34 ± 0.14	14.22 ± 2.19	61.26 ± 4.46	ND	ND	2x
1347	8.25 ± 0.1	13.75 ± 1.43	65.49 ± 10.92	8.15 ± 6.19	ND^**^	4x
1348	4.14 ± 0.03	25.87 ± 8.88	51.96 ± 1.79	0	ND^**^	2x
1515	3.81 ± 0.14	15.62 ± 2.2	60.97 ± 5	ND	ND	2x
1516	3.74 ± 0.08	15.09 ± 3.77	62.75 ± 5.41	ND	ND	2x
1722	8.41 ± 0.09	6.01 ± 1.05	69.33 ± 4.68	ND	ND	4x
1729	9.17 ± 0.21	4.95 ± 1.38	69.88 ± 4.45	79.88 ± 1.69	150.3± 25.2	4x
1736	8.28 ± 0.2	6.12 ± 1.43	74.32 ± 3.78	7.17 ± 4.5	91.5± 19.9	4x
1846	4.6 ± 0.27	15.85 ± 1.94	54.39 ± 1.85	6.85 ± 5.5	ND^**^	2x
2195	4.24 ± 0.1	14.57 ± 1.8	56.28 ± 3.21	ND	ND^**^	2x
2691	4.35 ± 0.11	13.11 ± 2.19	62.23 ± 4.63	ND	ND^**^	2x
2719	4.53 ± 0.19	13.64 ± 3.51	56.72 ± 3.25	0	76.1 ± 28.9	2x
2720	4.08 ± 0.18	14.63 ± 2.53	54.02 ± 3.75	5.46 ± 1.97	202.9 ± 79.5	2x
3081	8.52 ± 0.15	6.35 ± 1.26	70.79 ± 5.39	ND	103.8 ± 31.4	4x
3085	8.97 ± 0.41	5.54 ± 1.31	72.48 ± 4.55	70.37 ± 1.8	127.1 ± 75.4	4x
3099	9.16 ± 0.19	6.76 ± 1.89	75.51 ± 6.78	ND	146.8 ± 33.2	4x
3131	9.1 ± 0.29	6.47 ± 2.3	69.61 ± 4.43	83.01 ± 2.31	153.3 ± 56.3	4x

*ND, Not determined*.

♦ *Nuclear 2C DNA of the donor 90-002 was previously reported by Tel-Zur et al. (2011b)*.

** *Abortion during fruit maturation or less than three matured fruits*.

### Confirmation of gamete origin using SSR markers

A total of 23 pairs of SSR primers were used to flank the allelic sequences containing the microsatellites, and the band patterns of the putative gamete-derived lines and the donor species were compared. Twenty-one pairs of SSR primers showed a similar pattern (monomorphism), non-specific amplification, or amplification failure for *Hylocereus* (Table S1).

**Figure 1:**
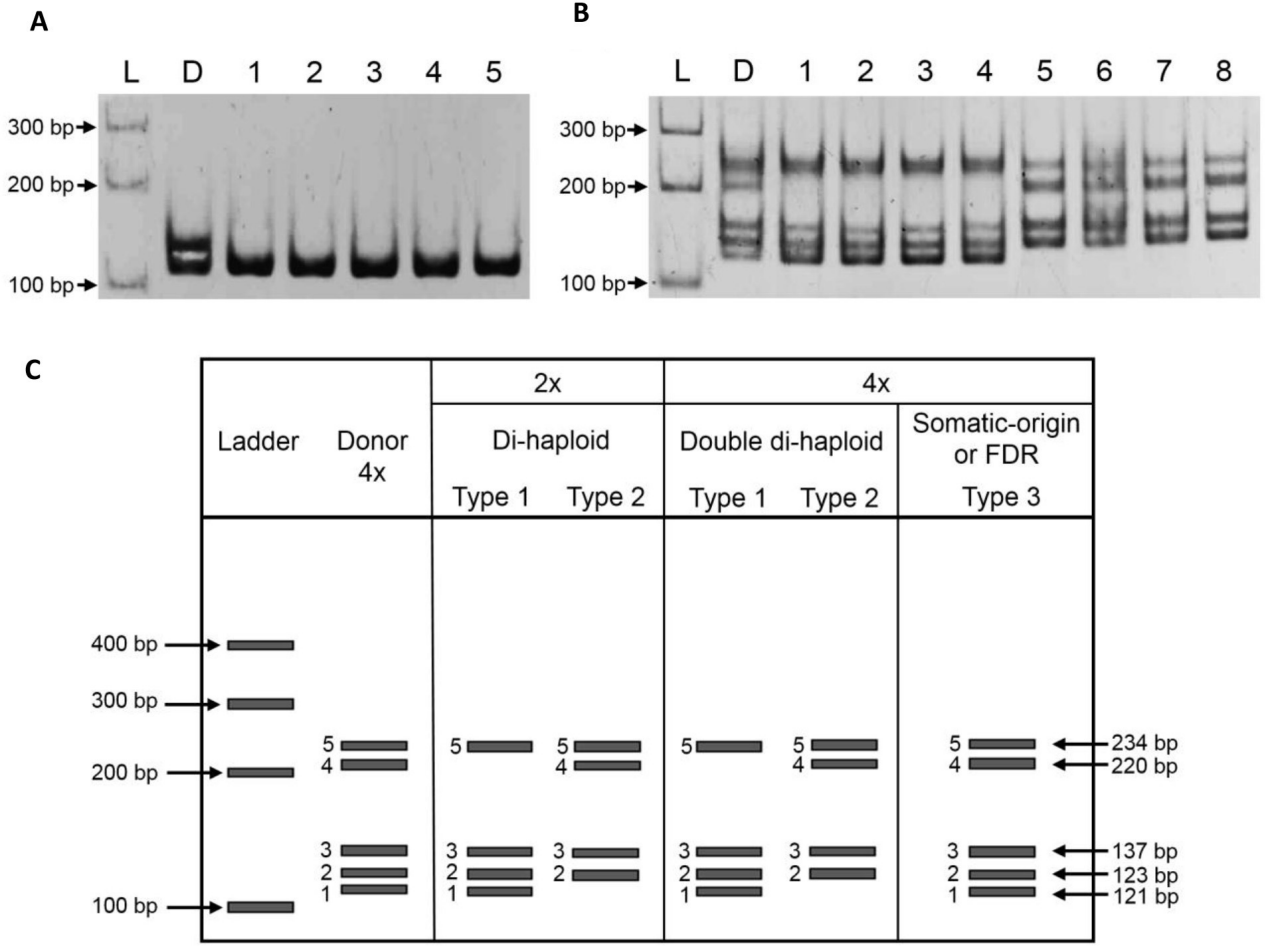
Polyacrylamide gel electrophoresis. **(A)** *H. monacanthus* amplified alleles from the microsatellite site *Pchi47*. Two alleles were observed in the diploid donor *H. monacanthus* but only one in the androgenic lines. Lane L: 100-bp DNA ladder; Lane D: donor plant *H. monacanthus*; Lanes 1–5: the resulting androgenic lines: 007 (1); 008 (2); 010 (3); 011 (4); and 7057 (5). **(B)** *H. megalanthus* amplified alleles from the microsatellite site *Pchi44*. Five alleles were observed in the tetraploid donor and somatic or FDR lines and four alleles were observed in the double di-haploid (gamete-derived) lines. Lane L: 100-bp DNA ladder; Lane D: donor plants (accessions 90-002 and 96-663); Lanes 1, 2, 3 and 7, 8: di-haploid gamete-derived lines; Lanes 4–6: double di-haploid (gamete-derived) lines, 432 (4); 1347 (5); 1736 (6). **(C)** Schematic analysis of band pattern types following amplification with *Pchi44* in *H. megalanthus*. Arrows indicate the positions and nucleotide lengths of the DNA bands.

Primer pair *Pchi47* successfully segregated a pair of heterozygous alleles in the diploid *H. monacanthus* donor (Figure 1A). The amplified bands from *H. monacanthus* were the 118- and 120-bp bands (Figure 1A, Table S2). Only one of the alleles (118 bp) was detected in all five androgenic lines, thus proving their male gamete origin. Multiple sequence alignment of alleles amplified from *H. monacanthus* and the cactus species *Polaskia chichipe* with primer pair *Pchi47* indicated different numbers of repeat motifs in the two species but a high identity of 92.5% (Table S2, Figure S1).

In *H. megalanthus*, only primer pair *Pchi44* showed polymorphic alleles between the donor species and the gamete-derived lines. The tetraploid donor *H. megalanthus* (accessions 90-002 and 96-663) exhibited five amplified allelic bands, but only four were observed in the 46 gamete-derived lines having about half of the total DNA content (Figure 1B). Three lines (432, 1347, and 1736) with a DNA content similar to that of the tetraploid donor species also shared only four bands, thereby proving their gamete origin. The other 21 lines with a DNA content similar to that of the donor species shared the same five-band pattern as the donor (Figures 1B,C). The five alleles were termed bands 1-5 according to their ascending nucleotide lengths, i.e., 121, 123, 137, 220, and 234 bp, respectively (Figure 1C). In summary, it was found that both the two donors and the tetraploid regenerants from *H. megalanthus* exhibited five bands (bands 1-5) at the SSR locus *Pchi44*, whereas the di-haploid and double di-haploid lines exhibited two types of four-band pattern, namely, type 1 (bands 1, 2, 3, and 5) or type 2 (bands 2, 3, 4, and 5) (Figure 1C). Accordingly, a tetraploid exhibiting the same band pattern as the di-haploids (type 1 or type 2) could be identified as a double di-haploid line (of gamete origin).

Full sequencing data for each allele revealed that the desired tandem repeat units and full primer sequences were present in all five alleles (Table S3). Multiple sequence alignment between the alleles from *H. megalanthus* and *P. chichipe* at the microsatellite site *Pchi44* revealed the highest similarity (98.5%) between band 3 from *H. megalanthus* and the amplified band reported in *P. chichipe* (GenBank number: AY147834; Otero-Arnaiz et al., 2004; Table S3, Figure S2).

### Cytological, histological and morphological evaluation

#### Hylocereus monacanthus double haploid lines

**Figure 2:**
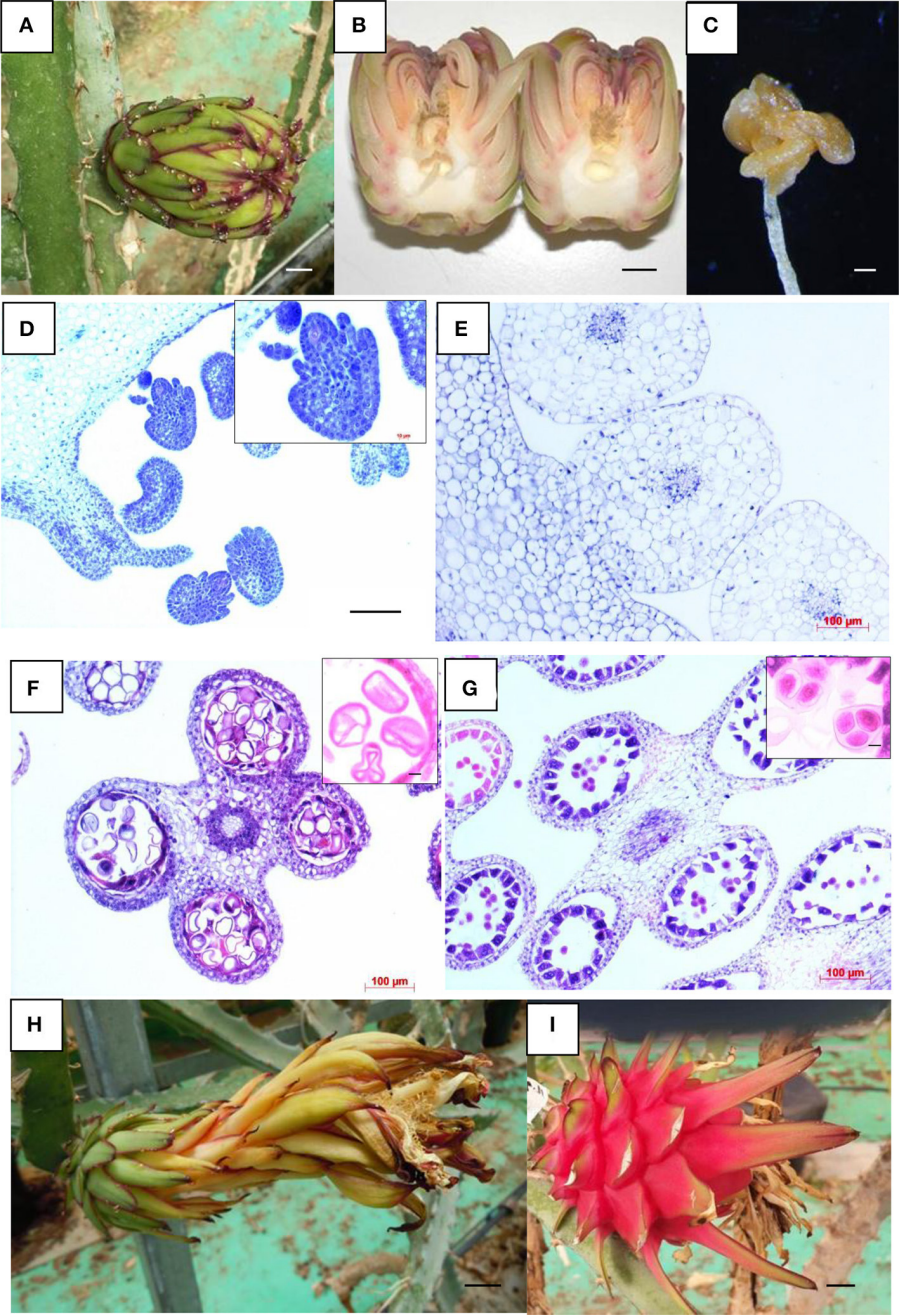
Reproductive parts of the diploid donor *H. monacanthus* and androgenic *H. monacanthus* lines. **(A)** Flower bud from the androgenic line 007, showing an atypical folded end (scale bar 1 cm). **(B)** Aborted open flower bud from the androgenic line 011 (scale bar 1 cm). **(C)** Deformed anther detached from an aborted flower bud of androgenic line 011 (scale bar 200 μm). **(D–G)** Histological sections of flower buds having the same length (6 cm), as follows: **(D)** Androgenic line 007 showing abnormal ovules (scale bar 100 μm), with a close-up of a shrunken ovule in the upper right-hand inset (scale bar 10 μm), **(E)** Well-developed ovules of the diploid donor (scale bar 100 μm), **(F)** Anther of the androgenic line 007 showing aborted tetrads, and a few uninucleate microspores with an atypical triangular shape (scale bar 100 μm), with a close-up of aborted pollen grains in the upper right-hand inset (scale bar 10 μm) **(G)** Anther of the diploid donor showing well-developed and viable tetrads (scale bar 100 μm), with a close-up of viable grains in the upper right-hand inset (scale bar 10 μm). **(H)** Anthesis in the androgenic line 011 (scale bar 1 cm), and **(I)** Mature fruit from line 011 (scale bar 1 cm).

Plantlets of the five androgenic *H. monacanthus* lines have been growing under greenhouse conditions since 2009 (Garcia et al., 2009a,b). These lines showed inferior characters, including very slow vegetative development and an extended juvenile stage of several years. The plants were similar to the donor species in terms of stem and spine shapes, but differed in floral development (Weiss et al., 1995; Nerd and Mizrahi, 2010). In the androgenic plants, the flower buds, which started to develop in October 2013 in two lines (007 and 011) and 1 year later in the other three, abscised before anthesis (Figures 2A,B). In the very early floral development stages, the five lines shared a similar flower bud morphology with that of the donor plant, i.e., green sepals with a red-violet border (Figure 2A). However, the superior part of the flower bud was abnormal, showing a flattened and folded open end (with the sepals folded inside), revealing the stigma. The pistil and anthers exhibited an atypical deformed shape (Figures 2B,C). Most of these flower buds dropped off before anthesis, obviating the possibility of studying pollen and ovule viability. Histological sections obtained during floral development (flower buds of 6 cm in length in line 007) showed an ovary that contained very small shrunken ovules with an abnormal shape (Figure 2D); in contrast, in the donor plant (having flower buds of similar length) the ovules were larger and exhibited a typical plump and globular shape (Figure 2E). Histological observations were also performed for the anthers of the line 007 flower buds described above. Those anthers were strikingly degraded, showing mostly aborted pollen grains with an atypical triangular shape and, in several parts, a folded-in tapetum (Figure 2F), while in the donor plant normal tetrads with the typical almost circular shape were observed (Figure 2G). In line 011, the single flower that developed fully to anthesis (Figure 2H) and set fruit in October 2016 (Figure 2I) produced a fruit that weighed 146.5 g. Fruit weight in the donor plant averages 355.0 ± 90.4 g.

#### Hylocereus megalanthus di-haploid and double di-haploid lines

**Figure 3:**
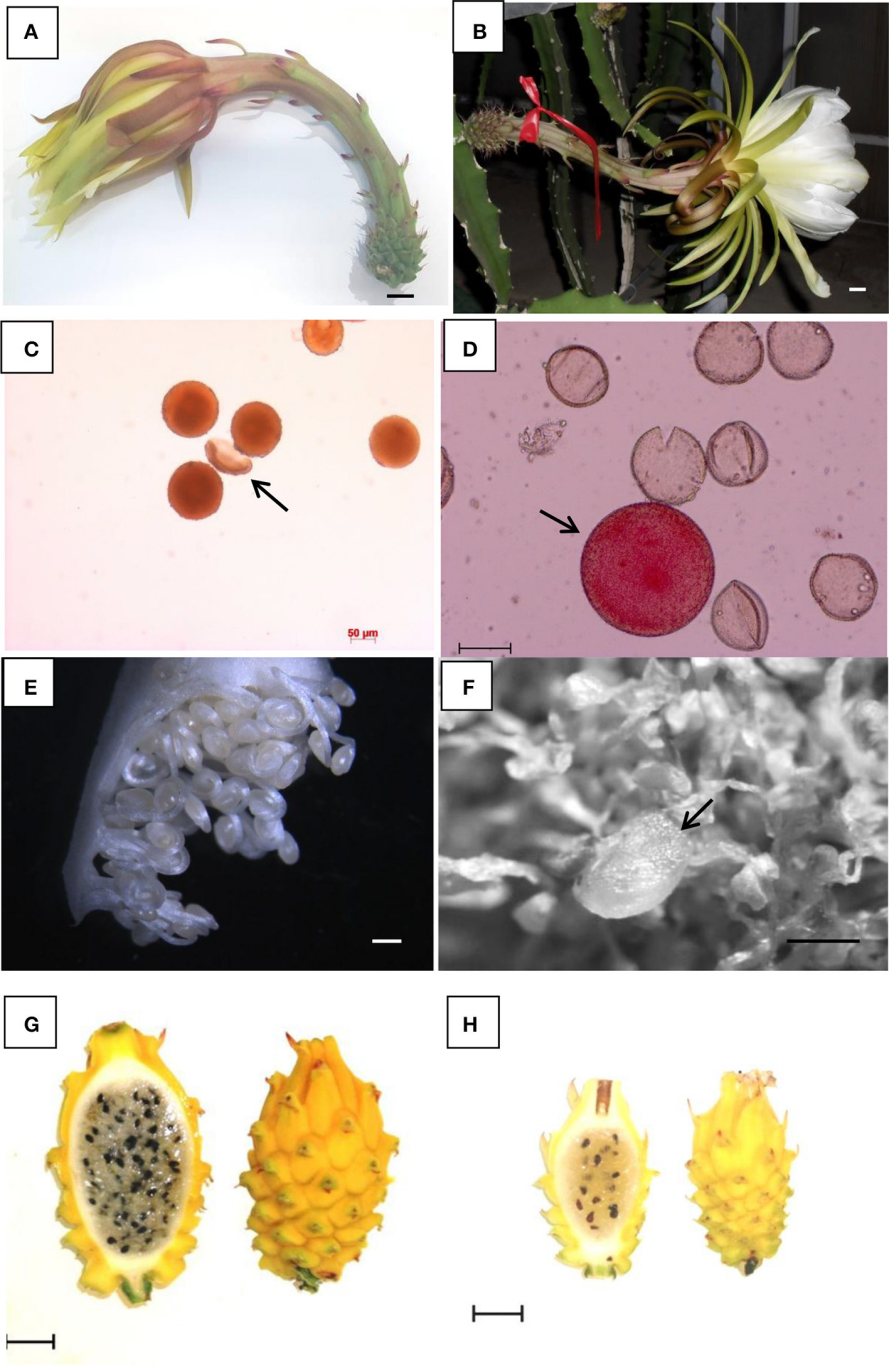
*H. megalanthus* **(A,C,E,G)** and gamete-derived *H. megalanthus* lines **(B,D,F,H)**. **(A,B)** Flower at anthesis in the donor plant and in the di-haploid line 74 (scale bar 1 cm). **(C,D)** Well-stained pollen grains with a normal subglobular shape and a single aborted grain (arrow) in the donor plant, and aborted (shrunken and unstained) pollen grains from the di-haploid line 1060 showing a single well stained grain (arrow) (scale bar 50 μm). **(E,F)** Ovules at anthesis in the donor plant (normal shaped) and in the double di-haploid line 1347 showing a single normal developed ovule (arrow) and several aborted ovules (scale bar 0.5 mm). **(G,H)** Mature fruit in the donor plant and in the di-haploid line 8, showing aborted (brown) and viable (black) seeds (scale bar 2 cm).

The vegetative development of the gamete-derived *H. megalanthus* lines was faster than that reported above for the androgenic *H. monacanthus*. These plantlets were also grown in the greenhouse from 2009, and they started to flower 1 year later. At anthesis, flowers reached an average length of 34.16 ± 3.05, 37.4 ± 0.66, and 41.58 ± 2.96 cm for the di-haploid, double di-haploid (determined only for lines 1347 and 1736) and tetraploid lines, respectively, with the flowers of the somatic tetraploid lines reaching a similar average length to that of the tetraploid donor plants (Weiss et al., 1995; Figures 3A,B). Statistically significant differences were found between flowers of the di-haploid lines and those of the tetraploids of somatic origin (*P* < 0.0001) (Figure S3). The other measured trait that showed differences between lines with different ploidy levels and different origins was the width of the flower bud (the widest part of the ovary) at anthesis, reaching 2.86 ± 0.33, 3.66 ± 0.18, and 3.40 ± 0.46 cm for the di-haploid, double di-haploid (determined only for lines 1347 and 1736) and tetraploid lines, respectively. In addition, for the flowers of the different lines, statistically significant differences were found between the di-haploid lines and the tetraploids of somatic origin (*P* < 0.002) and between the di-haploid lines and the tetraploids of gamete origin (*P* < 0.0062) (Figure S4). Finally, failure of anther dehiscence at anthesis was observed in all the di-haploid lines.

Pollen viability was 73.7 ± 1.46 and 70.2 ± 3.58% for the donor lines 90-002 and 96-663, respectively, and was very much lower in all the gamete-derived lines (Table 2, Figures 3C,D). As a result of the failure of anther dehiscence at anthesis, pollen from di-haploid lines was taken from excised anthers and examined. In nineteen di-haploid lines complete male sterility was observed (all the pollen grains observed under light microscopy were aborted), and a range of 3.69 ± 4.4 to 18.5 ± 3.16% was found in another seven di-haploid lines. In addition, extremely low pollen viability was observed in the double di-haploid lines (432, 1347, and 1736), i.e., 9.81 ± 6.20, 8.15 ± 6.19, and 7.17 ± 4.50%, respectively (Table 2), even though normal anther dehiscence at anthesis was observed in these lines.

Well-developed ovules were observed in the donor plant (Figure 3E). In the gamete-derived lines, most of the flowers aborted before anthesis or soon after pollination. The abscised flowers examined revealed that most of the ovules were abnormally shaped, and only few showed the typical form (Figure 3F). Average fruit weight for fourteen di-haploid lines varied from 30.6 ± 7.1 to 202.9 ± 79.5 g (Table 2). Statistically significant differences in fruit weight were found between the di-haploid lines and the tetraploids of somatic origin (*P* < 0.0125) and between the tetraploids of somatic origin and the tetraploids of gamete origin (*P* < 0.0083) (Figure S5). No significant differences were found in terms of fruit weight between all the lines of gamete origin (di-haploids and double di-haploid) (*P* < 0.1571) (Figure S5). Fruit weight in the tetraploid donor lines 90-002 and 96-663 was 130 ± 52 and 143 ± 57 g, respectively (Figure 3G, Table 2). Fruit weight was low in the double di-haploid lines, being 10.5 ± 0.5 and 91.5 ± 19.9 g for lines 432 and 1736, respectively. In the double di-haploid lines, aborted (brown) and viable (black) seeds were observed (Figure 3H). Line 1347 has not yet set fruit (Table 2).

### Correlation between stomatal characteristics and ploidy level

The diploid donor *H. monacanthus* exhibited a lower stomatal density and a higher stomatal length than the androgenic *H. monacanthus* double-haploid lines (Table 1), with statistically significant differences in these two parameters being observed between some of the androgenic lines and the donor plant (Table 1). However, since all five androgenic lines and the donor *H. monacanthus* are diploid plants, neither stomatal density nor stomatal length can be used as indicative measures of ploidy level.

**Figure 4:**
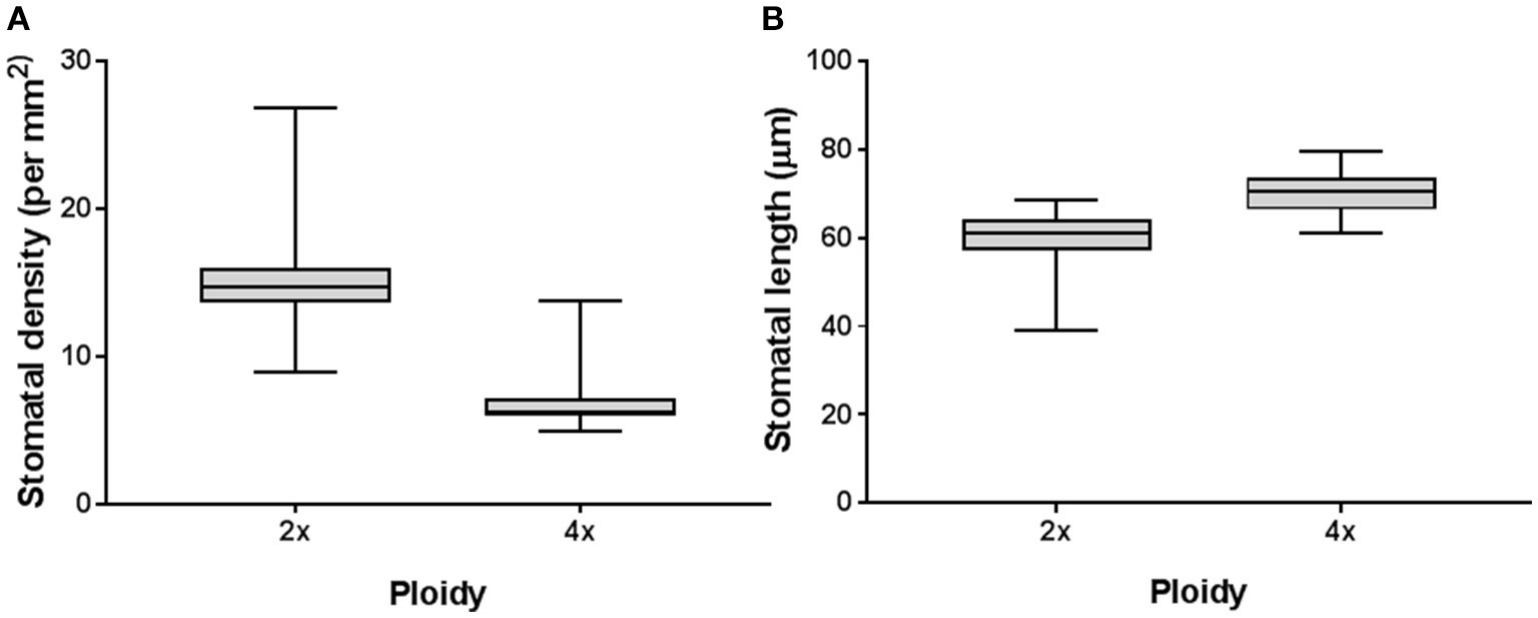
Putative gamete-derived *H. megalanthus* lines. All the di-haploid lines were regarded as one group, and all the tetraploid lines, as another group. **(A)** Association between DNA ploidy and stomatal density (*P*-value: < 0.0001, *F*-test *P*-value: 0.0036). **(B)** Association between DNA ploidy and stomatal length (*P*-value: < 0.0001, *F*-test *P*-value: 0.3071). Data was analyzed using unpaired *t*-test with Welch's correction.

For *H. megalanthus*, the di-haploids showed significantly higher stomatal density and a shorter stomatal length than the tetraploids (both at the level of *P* < 0.0001) (Figures 4A,B). For the di-haploids the average stomatal density was 15.17 ± 3.06 per mm^2^ [95% confidence interval (CI): 14.26–16.08 per mm^2^], and the average stomatal length was 60.01 ± 5.41 μm (95% CI: 58.41–61.63 μm), whereas for the tetraploid lines (except the double di-haploid line 1347) the values for average stomatal density and length were 6.81 ± 1.75 per mm^2^ (95% CI: 6.1–7.51 per mm^2^) and 70.22 ± 4.47 μm (95% CI: 68.41–72.02 μm), respectively.

## Discussion

In ovule or anther culture, plants can regenerate not only from gamete cells but also from somatic cells. Several plant species produce both reduced (*n*) and unreduced (*2n*) gametes with the somatic chromosome number (Bretagnolle and Thompson, 1995). Unreduced (*2n*) gametes (pollen or ovules) result from meiotic dysfunction, mainly as a result of two processes, namely, first division restitution (FDR) and second division restitution (SDR), which occur during abnormal first and second meiotic divisions, respectively (Bretagnolle and Thompson, 1995 and references therein). FDR gametes have two non-sister chromatids and exhibit levels of heterozygosity similar to those of the parent (maintaining all the parental genes except cross-over fragments), while SDR type gametes have two sister chromatids and exhibit lower levels of heterozygosity than those the parent (Bretagnolle and Thompson, 1995; Younis et al., 2014). Thus, a diploid status and a lower level of allelic diversity can be attributed either to an unreduced (*2n*) male gamete from SDR (the sister chromatids do not separate during the second meiotic division) or to spontaneous chromosome doubling in a haploid (*n*) male gamete, which restores the diploid status. However, for the diploid *H. monacanthus* there is evidence only of normal reduced gamete formation (Tel-Zur et al., 2003) and regular chromosome disjunction at anaphase I (Lichtenzveig et al., 2000), suggesting only a negligible occurrence of unreduced gametes in this species. Ploidy estimation (using flow cytometry analysis) for these five lines showed the same ploidy as the donor (Table 1). Nonetheless, these lines exhibited a lower level of allelic diversity in comparison with the donor plant (Figure 1A), and therefore they were identified as true androgenic double-haploid lines. Thus, we can assume that spontaneous chromosome doubling occurred during early plant development in all five androgenic *H. monacanthus* lines.

The tetraploid *H. megalanthus* is known to produce both normal reduced (*n*) gametes and unreduced (*2n*) gametes (Tel-Zur et al., 2003). Ploidy estimation for the putative gamete-derived *H. megalanthus* lines showed 46 diploids and 24 tetraploids (Table 2), where the diploids are indeed true gamete-derived lines (“di-haploid lines”). Among the 24 lines estimated as tetraploids, 21 lines showed the same five-band pattern as the donor line. This finding could indicate a somatic rather than a gamete-derived origin, but unreduced gametes of the FDR type possess levels of heterozygosity that are identical or close to identical with those of the parent. Furthermore, the origin of the three double di-haploid (432, 1347, and 1736) lines could derive either from a normal reduced gamete that had subsequently undergone spontaneous chromosome doubling or from unreduced gametes of the SDR type. Additional work using cytological, morphological and/or phenotypic markers—alongside molecular tools—is required to reveal the origin of these lines and to further examine their potential value for breeding.

On the basis of the general correlation between genome size (or ploidy) and stomatal parameters, extensive attempts have been made to develop methodologies for rapid pre-screening and ploidy estimation in plant species (see Germanà, 2011 and references within). In our hands, we found that stomatal density was a good proxy for the preliminary estimation of ploidy in gamete-derived *H. megalanthus* lines, since only few values were outliers or overlapped in the 2*x* and 4*x* results (Figure 4A). In contrast, considerable overlapping ranges in stomatal length were found between di-haploid and tetraploid regenerated lines (Figure 4B).

The five double-haploid *H. monacanthus* lines showed low vigor and developed abnormal flowers with malformed ovules and aborted pollen grains. Thus, the main obstacle to using double-haploid *H. monacanthus* lines in breeding programs is the severe male and female infertility of these lines, in keeping with the low fertility in double-haploid lines that has also been reported in other plant species. Joyce et al. (2003) suggested that under *in vitro* culture conditions a high level of mutations gives rise to deleterious characteristics. In a study on *Solanum*, low fertility was attributed to an aneuploid status, with 2C values deviating from those of the normal euploid (Valkonen et al., 1994). In robusta coffee (*Coffea canephora*), only half the double-haploid lines that had been planted out survived field conditions, and those lines showed low vigor and reduced fertility, suggesting a strong negative effect of homozygosity on vigor and reproductive characteristics (Lashermes et al., 1994). In apple (*Malus* × *domestica*), double-haploid lines also showed low vigor, together with compressed tree architecture and abnormal flower morphology (Höfer and Flachowsky, 2015). In double-haploid cocoa lines (*Theobroma cacao*), fertility varied from levels similar to those of the diploid donor to very low levels, which were not improved upon grafting (Lanaud, 1987). The reduced fertility in these cocoa lines, which was manifested in the abnormal development of female and male organs (as expressed by both pollen abortion and the lack of normal differentiation of embryo sacs), was attributed to the phenomenon known as “homozygote depression” that is forced in cross-fertilized species (Lanaud, 1987). Similar findings were obtained for our double-haploid *H. monacanthus* lines, i.e., low vigor and abnormal flower development.

The di-haploid and double di-haploid *H. megalanthus* lines were female fertile to different degrees (Table 2). Among the 46 di-haploid lines, 14 set fruit, with a wide range of average fruit weights between lines and a large standard deviation at the line level (Table 2); performances therefore ranged from “similar to donor lines” to “very low female fertility.” Reduced fruit weight was found in all the lines of gamete origin (di-haploids and double di-haploids) in comparison with the tetraploids of somatic origin (Figure S5). Some of these di-haploid and double di-haploid lines were controlled crossed with pollen donors from other *Hylocereus* species (interspecific crosses); fruits of these interspecific crosses reached maturity and contained viable seeds (data not shown). These new interspecific hybrids will be used for continuing the breeding program and further genetic studies. Although another 13 di-haploid lines did bloom (and pollen viability was measured), the developing fruits abscised before maturation (or set less than three fruits). Similar to the reduced fertility discussed above for *H. monacanthus*, reduced fertility in *H. megalanthus* might be linked to the abnormal ovules produced in the di-haploid and double di-haploid lines (Figure 3F). Thus, it is likely that for this tetraploid species, too, reduced heterozygosity drastically affected viability, supporting once more the theory of “homozygote depression.”

Male fertility (measured as the percentage of pollen grain viability) was low for all the di-haploid and double di-haploid *H. megalanthus* lines, i.e., from very low levels to totally sterile lines (Table 2). In haploid lines, chromosome doubling is needed to restore fertility (Germanà, 2011). However, in our hands, the three double di-haploid *H. megalanthus* lines and the above-described double-haploid *H. monacanthus* lines also showed very low level of pollen viability, pointing to the simple fact that doubling the set of chromosomes was not sufficient to restore fertility in gamete-derived *H. monacanthus* or *H. megalanthus* lines.

Knowledge about the origin of the species in any crop improvement program is fundamental to the success of the project. In this regard, our above-described findings support Bauer's taxonomy that placed the tetraploid *H. megalanthus* in the same taxon as the diploid *Hylocereus* species (Bauer, 2003). Further support comes from the study of Plume et al. (2013), which showed a low level of polymorphism within or among *H. megalanthus* accessions, meaning an autopolyploid rather than an allopolyploid origin. Their findings, in turn, are corroborated by the almost insignificant gametoclonal variation (namely, variation in plant morphology) observed in our study in the di-haploid *H. megalanthus* lines in comparison with the donor plant: the only difference lay in the size of the floral organs (bud length and width) of the di-haploid lines, which was smaller than that of the tetraploids (Figures S3, S4) These findings contrast with the wide variability in plant morphology reported for a di-haploid potato by Rousselle-Bourgeois and Rousselle (1992).

## Concluding remarks

Among the different facets of our long-term breeding and research program for expanding the cultivation of *Hylocereus*—as a new emerging fruit crop—an attempt was made to improve breeding efficiency by the development of gamete-derived lines in two *Hylocereus* species. The current study shows that the use of double-haploid *H. monacanthus* lines is severely limited by the sterility of these lines. Nonetheless, hybrid plants produced from the single fruit that was obtained from the interspecific cross of the double haploid *H. monacanthus* line 011 as the female parent are currently growing in our greenhouse, and additional efforts to obtain more progeny will be made in the near future. Hybrids from some of the di-haploid and double di-haploid *H. megalanthus* lines are also currently growing in our greenhouse. For these lines, the pollen grains were almost totally sterile, but the ovules showed different levels of fertility. Thus, in practice, these lines are male sterile (require cross pollination) but they are female fertile to different degrees and can therefore be used to develop new hybrids. Although, the number of produced hybrids was limited and probably lower than that desirable for intensive breeding, this new plant material offers unique possibilities for genetic research and breeding.

## Author contributions

DL and NT-Z conceived the experimental design, DL conducted the cytological and molecular assessments, MA performed the morphological evaluation, RS conducted the histological work, and DL and NT-Z wrote the manuscript. All authors read and approved the final manuscript.

### Conflict of interest statement

The authors declare that the research was conducted in the absence of any commercial or financial relationships that could be construed as a potential conflict of interest.

## Abbreviations

BGU: Ben-Gurion University of the Negev
CAM: crassulacean acid metabolism
FDR: first division restitution
sd: standard deviation
SDR: second division restitution
SSR: simple sequence repeat.
