# Supplementary material for: Homozygote Depression in Gamete-Derived Dragon-Fruit (Hylocereus) Lines
Source: Front Plant Sci. 2018 Jan 5;8:2142. doi: 10.3389/fpls.2017.02142 (PMC5760538; doi:10.3389/fpls.2017.02142)
Supplement: Supplementary file 1 [file DataSheet1.pdf]

TABLE S1 | Characteristics of SSR primers used in the analysis. Annealing temperature (T_a) and number of alleles (N_a) are indicated.

SSR locus	GenBank accession no.	Primer sequence (5'-3')	Repeat motif	T_a (°C)	Allelic size range (bp)	N_a	Primer origin (specie)	References	Results for *hylocereus*
AaA3	DQ314551	F: GCAAGCAAGAGTATGGTGAATTGG<br>R: AGTTATTTTCACGGTAACACACATGG	$(AAG)_{13}$	64 °C	138-168	10	*Astrophytum asterias*	Terry et al., 2006	No amplification
AaD9	DQ314553	F: CTGTTTAGTTCTCTCGTCTTCACC<br>R: CTCCGCTTTTACTGCTAGCACC	$(AG)_{10}$	64 °C	135-143	6			No amplification
AaG3	DQ314554	F: CTAACAGAGAATCCAAGGCTTTTCC<br>R: AATCGCCAGCCGAGGGAGAC	$(CA)_7$	64 °C	127-133	4			No amplification
mEgR17	AM746465	F: ATCGTTGAAAGAGGCGAAA<br>R: TCCCTCTTCTTCGTCAGAGC	$(AG)_{11}$	58 °C	330-347	4	*Echinocactus grusonii*	Hardesty et al., 2008	Non-specific amplification*
mEgR39	AM746463	F: GAGCGCAGAATTGAGGTG<br>R: GATGTGGCATTTTCAGAC	$(GA)_9CA(GA)_2$	58 °C	161-171	3			No polymorphism♦
mEgR76	AM746467	F: TCACAATTTGGAAGGAAGCA<br>R: GTGAGCAAAGGGCTGATTTC	$(AG)_{10}(AAG)_2C(CA)_2$	58 °C	376-396	7			No polymorphism
mEgR78	AM746469	F: AGCCCAAAGCCCAACTTATT<br>R: TGCATGCAATCATAAGGTTTTC	$(AG)_{13}GAG(CA)_3$	58 °C	148-242	2			No polymorphism
mEgR98	AM746470	F: ACCCTAGTGGGGTCGAGAAT<br>R: GTCGCCCAGAACCCTAGTCT	$(AG)_{12}AA(AG)_4$	58 °C	172-187	5			No polymorphism
Ops.9	EX720594	F: AACTGCCTCACACGAGTTCC<br>R: GCTACGAAATCTGCCGAGTC	$(TGA)_9$	53 °C	N/A	17	*Opuntia ficus indica*	Caruso et al., 2010	Non-specific amplification
Ops.24	EX720605	F: TCCTTCCATTTCCACCACAC<br>R: CAAGACCCCTCATTCCAAAG	$(CT)_{24}$	53 °C	N/A	18			Non-specific amplification
Pchi21	AY147837	F: CGTTTAGCCCCTCTTTCTCC<br>R: GTTCCCAACTGACCGACAAC	$(CT)_5(AT)_3(GT)_8GA(GT)_5$	60 °C	124	6	*Polaskia chichipe*	Otero-Arnaiz et al., 2004	Non-specific amplification
Pchi25	AY147836	F: GCCCTTCTAAGGCCATTTCT<br>R: ATTCCGTGTCAAGATTGTGC	$T_5(GT)_{16}A_5$	60 °C	273	5			Non-specific amplification
Pchi44	AY147834	F: ATTCAAACAGGCCCACACAG<br>R: GGGTGTTAGAAGGAATAATAGCTTG	$(CA)_{17}$	59 °C	137	4			Showing polymorphism in *H. megalanthus*
Pchi47	AY147832	F: GTCCTTGTGGCTAGCCCTTT<br>R: CCATTTCTCTCGCCATCTG	$(TG)_{15}$	60 °C	120	2			Showing polymorphism in *H. monacanthus*
Pchi54	AY147831	F: CCTTGAGCTTTGACATTGAGA<br>R: GGAAGGTTTTCATTGGATGAG	$(CA)_5CG(CA)_5TG(CA)_{22}(TA)_3$	60 °C	170	8			No polymorphism
Ppri02	KC349893	F: TTCCATCGTCCCCTCACTTA<br>R: CATTCACCACCGTGAACACT	$(AG)_{11}$	66 °C	115-119	2	*Pachycereus pringlei*	Flores et al., 2014	No amplification
Ppri03	KC349894	F: GGTGTTCCTCGCTCTCATTC<br>R: CTCGCAAATCCAAGCAAAAT	$(CT)_{11}$	65 °C	133-155	10			No amplification
Ppri05	KC349896	F: AAACTGCAGGTGTTTCAGGG<br>R: AATGAAGCGAAAGGAAGCAA	$(GTTT)_8$	61 °C	166-186	5			No amplification
Ppri06	KC349897	F: GCTCACGTTGGCAGATTTGT<br>R: GGTGATGACAAAAGGTTTTGC	$(AAAT)_6$	50 °C	138-146	3			No amplification
Ppri07	KC349898	F: TGGACTTCCAAGGGATAATGA<br>R: TCAACTCAAAGTGTCAGTGCTG	$(AAAT)_8$	59 °C	127-143	4			No amplification
Ppri08	KC349899	F: AATAGCGCATGCCTCAAAGG<br>R: CAATAGTCCAGAAATAGGTCAGGTCA	$(CT)_8(CTTT)_2$	66 °C	109-126	4			No amplification
Ppri09	KC349900	F: AAGAGACAGGCCCTGAGACA<br>R: TCGTAGGTTCCATCACCACA	$(TC)_{10}$	68 °C	119-140	5			No amplification
Pmac102	HQ667131	F: TCTATAAGTGCCGATGGATGC<br>R: CACACCTCACTCCCAACCTC	$(AG)_9$	54 °C	188-120	2	*Pilosocereus machrisii*	Perez et al., 2011	No amplification

* Amplifications that generated several unstable bands containing partial sequences of forward and/or reverse primers, and primer pairs that did not flank the desired repeat motif.

♦ Amplifications that generated the same band patterns.

TABLE S2 | DNA sequences of *H. monacanthus* alleles from the microsatellite locus Pchi47. Nucleotides that are underlined are sequences from forward and reverse primers. Nucleotides that are marked with a grey background are tandem repeat units.

Plant species	Band no./ GenBank no.	Allele size (bp)	DNA sequence (5'-3')	Repeat motif
H. monacanthus	Band 1	118	GTCCTTGTGGCTAGCCCTTTCGGATCATGTGTCAATCTAT GTTAAGGACTTCATTTGTTTGTGTGTCTATGTGCGTGTTT GTGTGGCATATCAATTTCTCAGATGGCGAGAGAAATGG	$(TG)_3$
	Band 2	120	GTCCTTGTGGCTAGCCCTTTCGGATCATGTGTCAATCTAT GTTAAGGACTTCATTTGTTTGTGTGTGTCTATGTGCGTGT TTGTGTGGCATATCAATTTCTCAGATGGCGAGAGAAATGG	$(TG)_4$
P. chichipe	AY147832	120	GTCCTTGTGGCTAGCCCTTTCGGATCGTGTGTCAATCTAT GTTAAAGACTTCATTTGTGTGTGTGTGTGTGTGTGTGTGT GTGTGGGGCATATCAATTTCTCAGATGGCGAGAGAAATGG	$(TG)_{15}$

TABLE S3 │DNA sequences of *H. megalanthus* alleles from the microsatellite locus Pchi44. Nucleotides that are underlined are sequences from forward and reverse primers. Nucleotides that are marked with a grey background are tandem repeat units.

Plant species	Band no./ GenBank no.	Allelic size (bp)	DNA sequence (5'-3')	Repeat motif
H. megalanthus	Band 1	121	ATTCAAACAGGCCCACACAGCTACATCAGCCTTTAATTTATGCCTCCCTT<br>CACACACACACACACAACACCAAAGGAATGAAAACAAAGAAATAAACAAG<br>CTATTATTCCTTCTAACACCC	$(CA)_8$
	Band 2	123	ATTCAAACAGGCCCACACAGCTACATCAGCCTTTAATTTATGCCTCCCTT<br>CACACACACACACACACAACACCAAAGGAATGAAAACAAAGAAATAAACA<br>AGCTATTATTCCTTCTAACACCC	$(CA)_9$
	Band 3	137	ATTCAAACAGGCCCACACAGCTACATCAGCCTTTAATTTATGCCTCCCTT<br>CACACACACACACACACACACACACACACACAACACCAAAGGAATGAAAA<br>CAAAGAAATAAACAAGCTATTATTCCTTCTAACACCC	$(CA)_{16}$
	Band 4	220	ATTCAAACAGGCCCACACAGACCCAGAAAATTGCAGTGCGCTCTCCTCAC<br>CGTCTAGCCAGCTACGTTGCTGTGCTTATGTGCTCACGCATGCCTTAAAG<br>CGCATATATTGAAACTCCTACATCAGCCTTTAATTTATGCCTCCCTTCAC<br>ACACACACACACACAACACCAAAGGAATGAAAACAAAGAAATAAACAAGC<br>TATTATTCCTTCTAACACCC	$(CA)_9$
	Band 5	234	ATTCAAACAGGCCCACACAGACCCAGAAAATTGCAGTGCGCTCTCCTCAC<br>CGTCTAGCCAGCTACGTTGCTGTGCTTATGTGCTCACGCATGCCTTAAAG<br>CGCATATATTGAAACTCCTACATCAGCCTTTAATTTATGCCTCCCTTCAC<br>ACACACACACACACACACACACACACACACAACACCAAAGGAATGAAAACAA<br>AGAAATAAACAAGCTATTATTCCTTCTAACACCC	$(CA)_{16}$
P. chichipe	AY147834	137	ATTCAAACAGGCCCACACAGCTACATCAGCCTTTAATTTGTGCCTCCCTT<br>CACACACACACACACACACACACACACACACAACACCAAAGGAATGAAAA<br>CAAAGAAGTAAACAAGCTATTATTCCTTCTAACACCC	$(CA)_{16}$

```
HM band1 GTCCTTGTGGCTAGCCCTTTCGGATCATGTGTCAATCTATGTTAAGGACTTCATTTGTT- 59
HM band2 GTCCTTGTGGCTAGCCCTTTCGGATCATGTGTCAATCTATGTTAAGGACTTCATTTGTTT 60
PC band  GTCCTTGTGGCTAGCCCTTTCGGATCGTGTGTCAATCTATGTTAAAGACTTCATTTGTGT 60
         ************************** ****************** ************

HM band1 -TGTGTGTCTATGTGCGTGTTTGTGTGGCATATCAATTTCTCAGATGGCGAGAGAAATGG 118
HM band2 GTGTGTGTCTATGTGCGTGTTTGTGTGGCATATCAATTTCTCAGATGGCGAGAGAAATGG 120
PC band  GTGTGTGTGTGTGTGTGTGTGTGTGGGGCATATCAATTTCTCAGATGGCGAGAGAAATGG 120
          ******* * **** **** **** **********************************
```

FIGURE S1 | Multiple sequence comparison of the SSR locus (Pchi47) in *H. monacanthus* (HM) and *P. chichipe* (PC). Sequences were aligned using the multiple alignment procedure of Clustal W provided by the program (http://www.ebi.ac.uk/Tools/msa/muscle/). Asterisks indicate sequence identity. Nucleotides that are marked with a grey background are tandem repeat units.

```
HM SSR Band1      ATTCAAACAGGCCCACACAG------------------------------  20
HM SSR Band2      ATTCAAACAGGCCCACACAG------------------------------  20
HM SSR Band3      ATTCAAACAGGCCCACACAG------------------------------  20
HM SSR Band4      ATTCAAACAGGCCCACACAGACCCAGAAAATTGCAGTGCGCTCTCCTCAC  50
HM SSR Band5      ATTCAAACAGGCCCACACAGACCCAGAAAATTGCAGTGCGCTCTCCTCAC  50
PC SSR Band       ATTCAAACAGGCCCACACAG------------------------------  20
                  ********************

HM SSR Band1      --------------------------------------------------  20
HM SSR Band2      --------------------------------------------------  20
HM SSR Band3      --------------------------------------------------  20
HM SSR Band4      CGTCTAGCCAGCTACGTTGCTGTGCTTATGTGCTCACGCATGCCTTAAAG  100
HM SSR Band5      CGTCTAGCCAGCTACGTTGCTGTGCTTATGTGCTCACGCATGCCTTAAAG  100
PC SSR Band       --------------------------------------------------  20


HM SSR Band1      -----------------CTACATCAGCCTTTAATTTATGCCTCCCTTCAC  53
HM SSR Band2      -----------------CTACATCAGCCTTTAATTTATGCCTCCCTTCAC  53
HM SSR Band3      -----------------CTACATCAGCCTTTAATTTATGCCTCCCTTCAC  53
HM SSR Band4      CGCATATATTGAAACTCCTACATCAGCCTTTAATTTATGCCTCCCTTCAC  150
HM SSR Band5      CGCATATATTGAAACTCCTACATCAGCCTTTAATTTATGCCTCCCTTCAC  150
PC SSR Band       -----------------CTACATCAGCCTTTAATTTGTGCCTCCCTTCAC  53
                                   ******************* *************

HM SSR Band1      ACACACACACACA----------------ACACCAAAGGAATGAAAACAA  87
HM SSR Band2      ACACACACACACACA--------------ACACCAAAGGAATGAAAACAA  89
HM SSR Band3      ACACACACACACACACACACACACACACAACACCAAAGGAATGAAAACAA  103
HM SSR Band4      ACACACACACACACA--------------ACACCAAAGGAATGAAAACAA  186
HM SSR Band5      ACACACACACACACACACACACACACACAACACCAAAGGAATGAAAACAA  200
PC SSR Band       ACACACACACACACACACACACACACACAACACCAAAGGAATGAAAACAA  200
                  *************                ********************

HM SSR Band1      AGAAATAAACAAGCTATTATTCCTTCTAACACCC  121
HM SSR Band2      AGAAATAAACAAGCTATTATTCCTTCTAACACCC  123
HM SSR Band3      AGAAATAAACAAGCTATTATTCCTTCTAACACCC  137
HM SSR Band4      AGAAATAAACAAGCTATTATTCCTTCTAACACCC  220
HM SSR Band5      AGAAATAAACAAGCTATTATTCCTTCTAACACCC  234
PC SSR Band       AGAAGTAAACAAGCTATTATTCCTTCTAACACCC  137
                  **** *****************************
```

FIGURE S2 │ Multiple sequence comparison of the SSR locus (Pchi44) in *H. megalanthus* (HM) and *P. chichipe* (PC). Sequences were aligned using the multiple alignment procedure of Clustal W provided by the program (http://www.ebi.ac.uk/Tools/msa/muscle/). Asterisks indicate sequence identity. Nucleotides that are marked with a grey background are tandem repeat units.

FIGURE S3 │Association between DNA ploidy and flower length. All the di-haploid lines were regarded as one group, the tetraploid of gamete origin, as a second group, and all the tetraploid lines of somatic origin, as a third group.

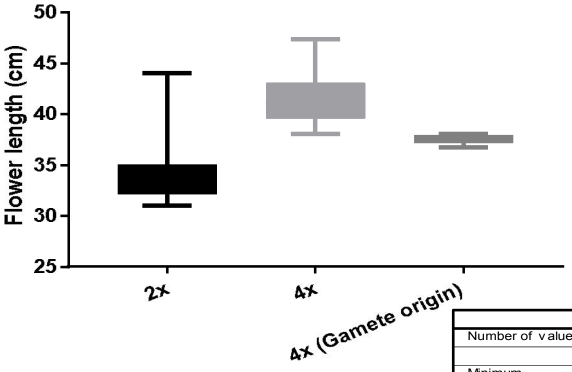

	2x	4x	4x (Gamete origin)
Number of values	16	13	3
Minimum	31	38	36.7
25% Percentile	32.13	39.5	36.7
Median	33.25	41	37.5
75% Percentile	35	43	38
Maximum	44	47.3	38
Mean	34.16	41.58	37.4
Std. Deviation	3.048	2.963	0.6557
Std. Error of Mean	0.762	0.8217	0.3786
Lower 95% CI	32.53	39.78	35.77
Upper 95% CI	35.78	43.37	39.03

Table Analyzed	flower length One-way ANOVA data				
Data sets analyzed	A : 2x	B : 4x	C : 4x (Gamete origin)		
ANOVA summary					
F	23.31				
P value	<0.0001				
P value summary	****				
Significant diff. among means (P < 0.05)?	Yes				
R square	0.6165				
Brown-Forsythe test					
F (DFn, DFd)	0.8103 (2, 29)				
P value	0.4546				
P value summary	ns				
Are SDs significantly different (P < 0.05)?	No				
Bartlett's test					
Bartlett's statistic (corrected)					
P value					
P value summary					
Are SDs significantly different (P < 0.05)?					
ANOVA table	SS	DF	MS	F (DFn, DFd)	P value
Treatment (between columns)	394.8	2	197.4	F (2, 29) = 23.31	P<0.0001
Residual (within columns)	245.6	29	8.468		
Total	640.4	31			
Data summary					
Number of treatments (columns)	3				
Number of values (total)	32				

Number of families	1							
Number of comparisons per family	3							
Alpha	0.05							
Tukey's multiple comparisons test	Mean Diff.	95.00% CI of diff.	Significant?	Summary	Adjusted P Value			
2x vs. 4x	-7.419	-10.1 to -4.736	Yes	****	<0.0001	A-B		
2x vs. 4x (Gamete origin)	-3.244	-7.765 to 1.278	No	ns	0.1968	A-C		
4x vs. 4x (Gamete origin)	4.175	-0.4276 to 8.778	No	ns	0.0811	B-C		
Test details	Mean 1	Mean 2	Mean Diff.	SE of diff.	n1	n2	q	DF
2x vs. 4x	34.16	41.58	-7.419	1.087	16	13	9.657	29
2x vs. 4x (Gamete origin)	34.16	37.4	-3.244	1.831	16	3	2.506	29
4x vs. 4x (Gamete origin)	41.58	37.4	4.175	1.864	13	3	3.168	29

FIGURE S4 | Association between DNA ploidy and flower width. All the di-haploid lines were regarded as one group, the tetraploid of gamete origin, as a second group, and all the tetraploid lines of somatic origin, as a third group.

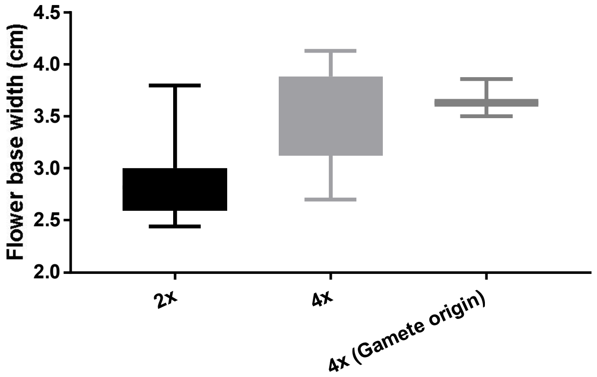

	2x	4x	4x (Gamete origin)
Number of values	16	13	3
Minimum	2.44	2.7	3.5
25% Percentile	2.595	3.125	3.5
Median	2.82	3.23	3.63
75% Percentile	3	3.885	3.86
Maximum	3.8	4.13	3.86
Mean	2.863	3.398	3.663
Std. Deviation	0.3265	0.4583	0.1823
Std. Error of Mean	0.08162	0.1271	0.1053
Lower 95% CI	2.689	3.121	3.21
Upper 95% CI	3.036	3.675	4.116

Table Analyzed	flower width				
Data sets analyzed	A : 2x	B : 4x	C : 4x (Gamete origin)		
ANOVA summary					
F	10.1				
P value	0.0005				
P value summary	***				
Significant diff. among means (P < 0.05)?	Yes				
R square	0.4105				
Brown-Forsythe test					
F (DFn, DFd)	1.378 (2, 29)				
P value	0.2681				
P value summary	ns				
Are SDs significantly different (P < 0.05)?	No				
Bartlett's test					
Bartlett's statistic (corrected)					
P value					
P value summary					
Are SDs significantly different (P < 0.05)?					
ANOVA table	SS	DF	MS	F (DFn, DFd)	P value
Treatment (between columns)	2.915	2	1.457	F (2, 29) = 10.1	P=0.0005
Residual (within columns)	4.186	29	0.1443		
Total	7.101	31			
Data summary					
Number of treatments (columns)	3				
Number of values (total)	32				

Number of families	1							
Number of comparisons per family	3							
Alpha	0.05							
Tukey's multiple comparisons test	Mean Diff.	95.00% CI of diff.	Significant?	Summary	Adjusted P Value			
2x vs. 4x	-0.536	-0.8863 to -0.1856	Yes	**	0.0020	A-B		
2x vs. 4x (Gamete origin)	-0.8008	-1.391 to -0.2105	Yes	**	0.0062	A-C		
4x vs. 4x (Gamete origin)	-0.2649	-0.8659 to 0.3361	No	ns	0.5287	B-C		
Test details	Mean 1	Mean 2	Mean Diff.	SE of diff.	n1	n2	q	DF
2x vs. 4x	2.863	3.398	-0.536	0.1419	16	13	5.343	29
2x vs. 4x (Gamete origin)	2.863	3.663	-0.8008	0.239	16	3	4.738	29
4x vs. 4x (Gamete origin)	3.398	3.663	-0.2649	0.2434	13	3	1.539	29

FIGURE S5 | Association between DNA ploidy and fruit weight. All the di-haploid lines were regarded as one group, the tetraploid of gamete origin, as a second group, and all the tetraploid lines of somatic origin, as a third group.

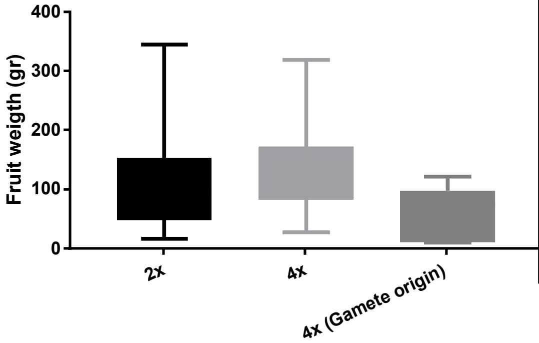

	2x	4x	4x (Gamete origin)
Number of values	90	88	8
Minimum	15.92	27.2	10.15
25% Percentile	47.51	82.13	10.51
Median	94.45	124.6	74.03
75% Percentile	152.9	171.8	97.13
Maximum	344.9	319.3	121.1
Mean	107.1	136.2	61.1
Std. Deviation	70.54	65.76	44.5
Std. Error of Mean	7.436	7.01	15.73
Lower 95% CI	92.32	122.2	23.9
Upper 95% CI	121.9	150.1	98.31

Table Analyzed	Fruit weigth				
Data sets analyzed	A : 2x	B : 4x	C : 4x (Gamete origin)		
ANOVA summary					
F	7.2				
P value	0.0010				
P value summary	***				
Significant diff. among means (P < 0.05)?	Yes				
R square	0.07295				
Brown-Forsythe test					
F (DFn, DFd)	0.9683 (2, 183)				
P value	0.3817				
P value summary	ns				
Are SDs significantly different (P < 0.05)?	No				
Bartlett's test					
Bartlett's statistic (corrected)	2.287				
P value	0.3187				
P value summary	ns				
Are SDs significantly different (P < 0.05)?	No				
ANOVA table	SS	DF	MS	F (DFn, DFd)	P value
Treatment (between columns)	65540	2	32770	F (2, 183) = 7.2	P=0.0010
Residual (within columns)	832917	183	4551		
Total	898457	185			
Data summary					
Number of treatments (columns)	3				
Number of values (total)	186				

Number of families	1							
Number of comparisons per family	3							
Alpha	0.05							
Tukey's multiple comparisons test	Mean Diff.	95.00% CI of diff.	Significant?	Summary	Adjusted P Value			
2x vs. 4x	-29.08	-52.98 to -5.185	Yes	*	0.0125	A-B		
2x vs. 4x (Gamete origin)	45.99	-12.82 to 104.8	No	ns	0.1571	A-C		
4x vs. 4x (Gamete origin)	75.08	16.21 to 133.9	Yes	**	0.0083	B-C		
Test details	Mean 1	Mean 2	Mean Diff.	SE of diff.	n1	n2	q	DF
2x vs. 4x	107.1	136.2	-29.08	10.11	90	88	4.067	183
2x vs. 4x (Gamete origin)	107.1	61.1	45.99	24.89	90	8	2.613	183
4x vs. 4x (Gamete origin)	136.2	61.1	75.08	24.91	88	8	4.262	183
